# Genetic diversity and evolutionary services for Nature‐based Solutions

**DOI:** 10.1002/ajb2.70238

**Published:** 2026-07-15

**Authors:** Alicia Mastretta‐Yanes

**Affiliations:** ^1^ Royal Botanic Gardens Kew, Richmond Surrey UK

**Keywords:** biodiversity, conservation, ecosystem services, Ecosystem‐based Adaptation, evolution, evosystem services, Global Biodiversity Framework, intraspecific diversity, socioecological challenges

From individuals to complex civilizations, humans have relied on nature for their well‐being through millennia, albeit reciprocity levels vary. In contemporary terms, and in the context of the most pressing issues humanity faces today, the “actions to protect, sustainably manage and restore natural and modified ecosystems in ways that address societal challenges effectively and adaptively, to provide both human well‐being and biodiversity benefits” are referred to as Nature‐based Solutions (NbS, Figure 1) (Cohen‐Shacham et al., 2016). NbS is an active field of socioenvironmental research with real‐world applications sitting at the intersection of people, nature, and environmental issues. For example, a successful NbS story is the community‐based mangrove restoration, which contributed to disaster risk reduction, protected key habitats, and increased local economy income from fisheries (see more at https://casestudies.naturebasedsolutionsinitiative.org/case-search/).

**Figure 1:**
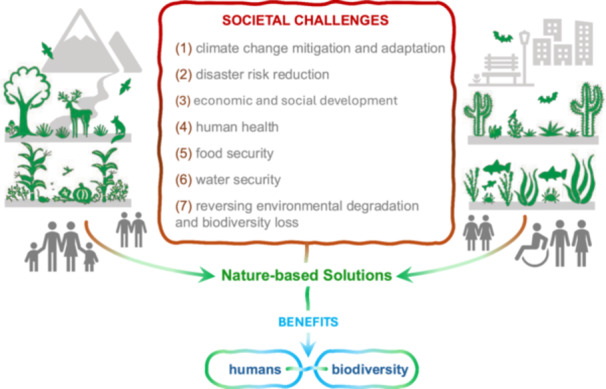
Nature‐based Solutions (NbS) are actions made by humans to protect, sustainably manage and restore natural and modified ecosystems in ways that address societal challenges effectively and adaptively, to provide both human well‐being and biodiversity benefits. According to the IUCN there are seven major societal challenges that can be addressed by NbS.

According to the International Union for Conservation of Nature (IUCN) there are seven major societal challenges that NbS can address (Cohen‐Shacham et al., 2016; Figure 1), including topics like disaster risk reduction, food security, climate change, and biodiversity loss. However, the concept of NbS has also sparked debate. On one hand, NbS are sensitive to the influence of *dominant framings* (see Box 1 (Glossary)) and power‐inequalities. For instance, certain framings can reinforce the human‐nature dichotomies or frame disagreements as undesirable (Hafferty et al., 2025). Therefore, they could be used for *greenwashing*, in top‐down approaches that commodify nature and can even harm biodiversity and human communities (Seddon, 2022). On the other hand, supporters of NbS maintain that beyond its original technical definition, NbS should be understood as ways of working with, and as part of, nature and framed to ensure that multiple values of nature are respected (Seddon, 2022); following guidelines to prevent the misuse of NbS (+ https://www.naturebasedsolutionsinitiative.org; IUCN, 2020). Furthermore, NbS are commonly supported and implemented by governments and civil organizations, and they are embedded in international agreements. Namely, NbS are explicitly or indirectly mentioned in several targets (https://www.cbd.int/gbf/targets) of the *Global Biodiversity Framework* (GBF), are included as part of the response options that advance multiple *Sustainable Development Goals* (IPBES, 2024), and they have been debated as part of the Convention on Climate Change. This signals that NbS are shaping real‐world decisions, thereby calling for rigorous research, which more explicitly should both: (1) include questions about how sustainability transformations are co‐produced, whose values and livelihoods are recognised, who decides how (and if) to undertake NbS, and who benefits from them (Hafferty et al., 2025); and (2) perform robust science across the different social and biological aspects of NbS.

Box 1 **Glossary**

**Access and Benefit‐Sharing (ABS):** An international framework that ensures equitable sharing of benefits arising from the use of genetic resources, particularly benefiting source countries and indigenous communities.

**Census population size (Nc):** The number of (often mature adult) individuals in a population. It is a key parameter for ecology that affects many demographic processes within and among populations (e.g., density dependence, birth and death rates, migration, competition, predator–prey interactions).

**Convention on Biological Diversity (CBD):** An international treaty for the conservation and sustainable use of biodiversity. The CBD was opened for signature in 1992 at the United Nations Earth Summit. Today, the Convention has been ratified by 196 parties.

**Digital Sequence Information (DSI):** A term currently being discussed in international policy referring to digital, publicly accessible data derived from genetic resources (DNA, RNA, proteins, and metabolites) of plants, animals, and microorganisms. It encompasses genetic information used in research for agriculture, medicine, and conservation, without requiring physical access to the organism.

**Dominant Framings:** The prevailing ways in which a concept, issue, or problem is understood, interpreted, and communicated within a particular discourse or field.

**Effective population size (Ne):** a concept from population genetics theory that measures the rate of loss of genetic diversity due to the random effects of genetic drift. Whereas Nc shapes the ecological dynamics of a population, Ne captures its evolutionary dimensions. Contemporary Ne in practice is related to how many individuals reproduce each generation, how related they are, and the proportion of males and females.

**Genetic drift:** The random change in the genetic composition of a population over time (generations). Genetic drift can result in differences in genetic composition between populations that are the result of chance, rather than adaptation to the local environment.

**Gene flow:** The exchange of genetic material between two populations, through migration of gametes (e.g., pollen) or individuals (e.g., offspring) from one population into another. For gene flow to occur, individuals entering the population must contribute genes to the population, i.e., reproduce and create offspring within that population.

**Global Biodiversity Framework (GBF):** A landmark agreement aiming to halt and reverse biodiversity loss by 2030 and achieve living in harmony with nature by 2050. It serves as the primary implementation mechanism for the CBD, setting 4 goals for 2050 and 23 action‐oriented targets for 2030.

**Greenwashing:** The practice of making deceptive claims or presenting a false image of environmental responsibility misleading the consumers, investors and general public to believe that a company or other entity is doing more to protect the environment than it is. It promotes false solutions that distract from and delay concrete and credible actions.

**Mutation:** A change in the DNA sequence of an organism. It is a source of new genetic diversity, which can be either beneficial, harmful or neutral.

**Standing genetic diversity:** The set of existing genetic diversity already present within a population's gene pool, rather than new mutations.

**Sustainable Development Goals (SDGs):** 17 goals adopted by the United Nations in 2015 as a universal call to action to end poverty, protect the planet, and ensure that by 2030 all people enjoy peace and prosperity

**Selection:** Variation in reproductive success associated with genetic variation. Selection can be natural, when the selective pressure comes from an element of nature (e.g., temperature, pathogens) or artificial, when the selection is made by humans (e.g., color preference).

Biodiversity is the foundation of NbS. It encompasses genetic, species, and ecosystem levels (Figure 2), yet there is a gap regarding both the degree and approaches by which genetic diversity (Figure 2) has been incorporated. For instance, the IUCN Global Standard for NbS (IUCN, 2020) does not mention genetic diversity at all, neither as a desired outcome or as a potential source of solutions. Omitting genetic diversity from desired outcomes undervalues its intrinsic value, neglecting that genetic diversity deserves to be part of a nature‐positive future by its own right (O'Brien et al., 2025), which is additionally problematic for two reasons.

**Figure 2:**
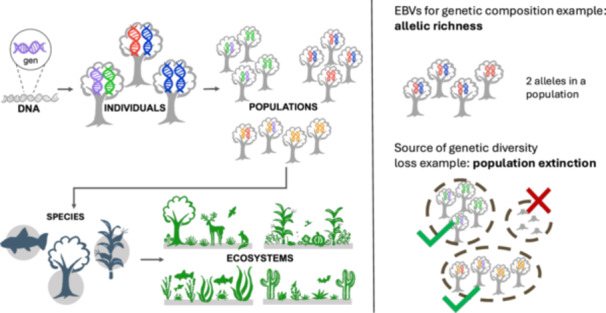
Genetic diversity as part of biodiversity. (A) Genetic diversity is the variation at the DNA level, it includes differences among individuals within populations of a single species, as well as differences between populations of it. Genetic, species and ecosystem diversity are the three levels of biodiversity recognized by the Convention on Biological Diversity. (B) Several metrics of genetic diversity exist and are part of the Essential Biodiversity Variables (EBVs) for genetic composition. A basic measurement of genetic diversity are the different alleles (a variant in the DNA sequence at a given gene) depicted here with different colors. A gold standard is to measure genetic diversity using sequencing methods, but it can also be monitored to some extent using proxies without DNA‐based data by focusing on threats to genetic diversity (O'Brien et al., 2022). For example, local population extinction is a source of genetic diversity loss, since all the unique alleles present in that population would also disappear (Hoban et al., 2024).

First, underrepresenting genetic diversity in NbS misses opportunities to enhance ecosystem resilience. A recent review (Dunlop et al., 2024), found that there is a cluster of research focused on genetic diversity that addresses the societal challenge of “reversing environmental degradation and biodiversity loss,” for instance by considering the effects of seed sourcing on restoration (Dunlop et al., 2024). This is important because genetic diversity is crucial to allow restored sites to continue to adapt to future environmental changes (Ngeve, 2025). However, genetic diversity has also other applications. In fact, all three main categories of Nature's Contributions to People (material, non‐material, and regulating) have been shown to be supported by genetic diversity (Des Roches et al., 2021).

Examples of the contributions of genetic diversity range from the more obvious link of genetic diversity as a source of variation for breeding or medical compounds, to less noticeable roles (Des Roches et al., 2021). For example, some riparian fungi are vital to riparian food webs and water quality; in one of such species, genetic diversity enhanced its decomposition capacity, especially under polluted conditions (Duarte et al., 2019), thereby showing that genetic diversity can influence water quality. Genetic diversity can also help ecosystems to withstand the conditions that climate change is already imposing. For instance, heat‐tolerant corals found in a natural population are now being used to grow bleaching‐resistant corals (Morikawa and Palumbi, 2019). Therefore, beyond reversing environmental degradation and biodiversity loss, incorporating genetic diversity to NbS not only could address other societal challenges, such as water security and climate change as shown by the examples above, but also, food security, human health, climate change, and even disaster risk‐reduction, as discussed elsewhere (Des Roches et al., 2021).

Second, limiting the approaches by which genetic diversity is included fails to recognize the role that some people have in supporting evolution for the mutual benefit of humans and nature. While the research opportunities for the role of genetic diversity in NbS may be many, we should be wary of a potential bias on how it has been applied so far. The largest body of research is probably the one that focuses on identifying the specific genetic variants responsible for a given adaptive trait, to then use them in breeding programs. I call this “the gene as a solution” approach. For instance, it is common to first screen crop landraces diversity, to then incorporate useful alleles into commercial varieties (e.g., Cheng et al., 2024). Similar approaches are starting to be used in wild species, both for commercial and restoration purposes. It is in this context that discussions surrounding genetic resources, *Access and Benefit‐Sharing*, and *Digital Sequence Information* arose (Sirakaya, 2022). While using genetic diversity in this way is valid and useful in some contexts, it does not account for where and how genetic diversity initially emerges. It also treats genetic diversity as a static attribute, while genetic diversity is probably the most dynamic component of biodiversity.

Changes in the genetic composition of a population can occur at human timescales by four evolutionary forces, i.e., *mutation, gene flow, selection* (natural or artificial), and *genetic drift*. For example, in the European ash, natural selection has led to significant changes in allele frequencies across thousands of genes in response to an invasive fungal pathogen (Metheringham et al., 2025). Although several factors influence the interplay of these forces, the main factors known to positively affect adaptive evolution are a large *effective population size*, high *standing genetic diversity*, and environmental change (Futuyma et al., 2023). Thus, genetic diversity is the dynamic outcome of evolution. It is a process, rather than a static attribute that maintains and creates options for adaptation to the future; conditions we have not yet accounted for. Maintaining and creating such options is not encompassed by ecosystem services, but by “evolutionary” (a.k.a. “evosystem”) services, i.e., the benefits from biodiversity that are derived from evolutionary processes in the past, present, and future, including novel adaptations (Faith et al., 2017).

The dynamism that sits at the core of evolutionary services is critically relevant for NbS, i.e., some groups of people (e.g., a local community that manages a forest) manage nature in a way that foments genetic diversity more than others (Mastretta‐Yanes et al., 2024). This is more evident in agroecosystems. For instance, traditional (*campesino*) maize farmers from Mexico save and use their own seeds, as opposed to buying improved—but more genetically homogeneous—commercial varieties. Collectively, they generate a large (about 500 million) effective population size that not only accounts for most of the extant genetic diversity of maize in North America, but that also increases the net number of new mutations (Bellon et al., 2018). Similarly, human actions also influence evolutionary processes of wild species depending on how ecosystems are managed. For example, hedgerows in agricultural landscapes serve as corridors for gene flow, which helps to maintain genetic diversity and resilience of forest patches (Van Geert et al., 2010). Thus, positive genetic outcomes can be obtained by undertaking ecological strategies that account for genetic principles, e.g., maintaining or improving connectivity or supporting large viable populations. Yet, the people that manage ecosystems in ways that already produce evolutionary services are seldom recognized, as their values and needs are very often ignored by public policy and, not surprisingly, they tend to coincide with the more vulnerable communities (Mastretta‐Yanes et al., 2024).

In conclusion, there is important potential for integrating genetics into NbS beyond current applications. Maximizing this potential requires both an evolutionary framework and acknowledgement of the values, needs, and livelihoods of communities that currently support evolutionary services. It also opens up exciting research opportunities at the intersection of evolution, genomics, ecology, and social sciences, including: (1) assessing the extent to which genetic diversity confers ecosystem resilience and other potential benefits; (2) exploring how NbS can be designed to deliver genetic and social benefits through ecological strategies; (3) identifying socio‐ecological systems that produce and maintain evolutionary services; and (4) exploring ways to support the values, incentives, and structures that enhance these systems.

## AUTHOR CONTRIBUTIONS

**A.M.‐Y.**: Conceptualization; Formal analysis; Investigation; Writing—original draft.
